# Characterizing Sympathetic Neurovascular Transduction in Humans

**DOI:** 10.1371/journal.pone.0053769

**Published:** 2013-01-10

**Authors:** Can Ozan Tan, Renaud Tamisier, J. W. Hamner, J. Andrew Taylor

**Affiliations:** 1 Cardiovascular Research Laboratory, Spaulding Rehabilitation Hospital, Boston, Massachusetts, United States of America; 2 Department of Physical Medicine and Rehabilitation, Harvard Medical School, Boston, Massachusetts, United States of America; 3 Sleep Laboratory and EFCR, Department of Rehabilitation and Physiology, University Hospital Grenoble, France; 4 HP2 Laboratory (Hypoxia: Pathophysiology) INSERM U1042 Joseph Fourier University, Grenoble, France; Idaho State University, United States of America

## Abstract

Despite its critical role for cardiovascular homeostasis in humans, only a few studies have directly probed the transduction of sympathetic nerve activity to regional vascular responses – sympathetic neurovascular transduction. Those that have variably relied on either vascular resistance or vascular conductance to quantify the responses. However, it remains unclear which approach would better reflect the physiology. We assessed the utility of both of these as well as an alternative approach in 21 healthy men. We recorded arterial pressure (Finapres), peroneal sympathetic nerve activity (microneurography), and popliteal blood flow (Doppler) during isometric handgrip exercise to fatigue. We quantified and compared transduction via the relation of sympathetic activity to resistance and to conductance and via an adaptation of Poiseuille’s relation including pressure, sympathetic activity, and flow. The average relationship between sympathetic activity and resistance (or conductance) was good when assessed over 30-second averages (mean *R^2^* = 0.49±0.07) but lesser when incorporating beat-by-beat time lags (*R^2^* = 0.37±0.06). However, in a third of the subjects, these relations provided relatively weak estimates (*R^2^*<0.33). In contrast, the Poiseuille relation reflected vascular responses more accurately (*R^2^* = 0.77±0.03, >0.50 in 20 of 21 individuals), and provided reproducible estimates of transduction. The gain derived from the relation of resistance (but not conductance) was inversely related to transduction (*R^2^* = 0.37, *p*<0.05), but with a proportional bias. Thus, vascular resistance and conductance may not always be reliable surrogates for regional sympathetic neurovascular transduction, and assessment from a Poiseuille relation between pressure, sympathetic nerve activity, and flow may provide a better foundation to further explore differences in transduction in humans.

## Introduction

Sympathetic nervous outflow is tonically active to support resting vascular tone [1], [2], and is rapidly increased to maintain pressure when systemic flow is reduced (e.g., when standing up) [3], [4]. Thus, it is critical to the control of arterial pressure, both on a moment-by-moment basis and in response to physiologic stressors, and so measurement and analysis of sympathetic activity has been a main focus of human cardiovascular research. Animal models point to a significantly nonlinear and nonstationary relation between sympathetic nervous outflow and vascular responses, though different animal models show marked differences in the dynamics of this relation [5]–[8]. Animal work does provide an excellent foundation from which to examine this relation, but data from quadrupeds is not sufficient to understand the quantitative relation in bipedal humans. Unfortunately, despite the importance of sympathetic neurovascular control for homeostasis [9], quantitative assessment of transduction of sympathetic nerve activity into a vascular response has rarely been pursued in humans.

In a novel approach, Stauss et al. [10] used median nerve stimulation to explore regional skin blood flow responses, and showed that blood flow decreases in a graded manner at stimulation rates between 0.075 and 0.1 Hz. However, the skin usually plays little role in arterial pressure control, whereas the renal and muscle vascular beds are dominant sites for control of systemic vascular resistance [11]. Unfortunately, renal sympathetic nerve recordings in humans are not feasible and muscle sympathetic nerve stimulation results in muscle contractions that alter regional blood flow independent of sympathetic effects [11]. Instead, isometric handgrip exercise has been used to derive a relation wherein progressive, exercise-mediated increases in peroneal nerve muscle sympathetic activity account for a majority of the progressive increases in vascular resistance [12]. However, it remains unclear which vascular response better reflects the physiology underlying regional sympathetic transduction - regional vascular resistance (the ratio of pressure over flow) or regional vascular conductance (the ratio of flow over pressure). For example, Kamiya et al. [13] used handgrip exercise to explore the effect of cardiovascular deconditioning on sympathetic neurovascular transduction. They assessed peroneal nerve muscle sympathetic activity and limb blood flow, and derived regional sympathetic transduction from relations between sympathetic activity and both vascular resistance and vascular conductance. After bed rest, regional sympathetic neurovascular transduction was augmented when assessed as the relationship of sympathetic activity to resistance, but when examined as the relationship to conductance, transduction was unchanged. Although the case has been made that conductance reflects vascular tone better than resistance when there are primarily changes in flow and not pressure [14], [15], one might not need to derive either resistance or conductance to assess regional vascular responses. That is, given our ability to obtain beat-by-beat changes in pressure, sympathetic activity, and flow, one might exploit these concurrent and independent measures to obtain an estimate of regional sympathetic neurovascular transduction. This approach is routinely used in animal models exploring the direct relation between stimulation of sympathetic nerves and blood flow responses [6], [7].

The importance of choosing a correct approach to quantify regional sympathetic neurovascular transduction may be best exemplified in the literature exploring the effects of elevated sympathetic activity with human aging. On one hand, sympathetic (alpha-adrenergic) blockade increases limb conductance (and decreases resistance) more in older individuals [16], proportional to resting sympathetic activity [17], suggesting a maintained neurovascular transduction with age. On the other hand, greater resting sympathetic activity relates to greater vascular resistance in young, but not in older men [18], indicating a potential reduction in neurovascular transduction with age. In fact, *in vivo* [19], [20] and *in vitro* [21], [22] contractile responses of human arteries to noradrenaline and tyramine are reduced with age, indicating a clear decline in regional sympathetic neurovascular transduction. Therefore, it remains unclear whether resistance or vascular conductance better reflects the impact of advancing age on the regional vascular responses.

We sought to assess the utility of different approaches to regional sympathetic neurovascular transduction in humans. We used isometric handgrip exercise as a physiologic stimulus to elicit progressive increase in sympathetic activity. We quantified and compared regional neurovascular transduction via three approaches: the relation of sympathetic activity to vascular resistance, the relation of sympathetic activity to vascular conductance, and an adapted Poiseuille relation between arterial pressure, sympathetic activity, and blood flow. As a physiologic check, we also determined whether the age-related differences in regional sympathetic neurovascular transduction reflects the previously reported decline in vascular contractile responses.

Our results show that the strength of the relation between increasing sympathetic nerve activity and regional vascular resistance or conductance can be highly variable, whereas a Poiseuille relation between arterial pressure, sympathetic nerve activity, and blood flow consistently captures a majority of this relationship. Thus, vascular resistance and conductance are not always reliable surrogates for regional vascular responses, whereas assessment from a Poiseuille relation may provide a foundation to further explore regional sympathetic neurovascular transduction in humans. We also found that sympathetic neurovascular transduction is attenuated in older compared to younger men.

## Materials and Methods

### Ethics Statement

All volunteers gave their written informed consent to participate. All protocols were approved by either the ethical committee at the Grenoble University Hospital Center or institutional review board at Spaulding Rehabilitation Hospital, and conformed to the Declaration of Helsinki.

### Subjects

10 young (26±1 years old) and 11 older (52±3 years old) men participated in this study (Table 1). All studies were conducted between 8 am and 12 pm, and all subjects had abstained from caffeine containing beverages for at least twelve hours, and from alcoholic beverages and exercise for at least 48 hours prior to the study. All subjects had normal body-mass index (19.9–29.9), were non-smokers, and free of elevated resting blood pressures and overt autonomic or cardiovascular diseases. None of the participants were using prescription medications.

**Table 1:** Resting hemodynamic variables.

	Younger	Older
Age*	26±1	52±3
BMI	22.4±1.0	23.7±0.8
Heart rate (bpm)	61±3	59±2
Systolic blood pressure (mmHg)	117±4	125±6
Diastolic blood pressure (mmHg)	66±3	76±4
Mean blood pressure (mmHg)	83±3	92±5
Flow velocity (cm/s)*	12.5±1.9	6.7±0.8
Vascular resistance*	8.3±1.3	17.3±2.2
Vascular conductance*	0.16±0.03	0.07±0.01
Muscle sympathetic nerve activity		
Spikes/min*	347±34	521±72
Spikes/100 hb*	574±57	882±118
Bursts/min*	12.2±1.5	20.6±2.5
Bursts/100 hb*	20.4±2.0	34.8±4.2

* *p*<0.05.

### Measurements and Protocol

Standard three-lead electrocardiogram and photoplethysmographic arterial blood pressure waveforms in a finger (Finapres, Ohmeda, Louisville, CO) were recorded continuously throughout the study. There is no systematic difference between intra-arterial and photoplethysmographic finger pressure measurements [23], and the latter has been shown to be suitable to track relative changes in arterial blood pressure during isometric handgrip exercise [24]. Oscillometric brachial pressures were recorded at 2 min intervals as a check for photoplethysmographic finger pressures throughout the study session.

Multiunit postganglionic muscle sympathetic activity was recorded from the peroneal nerve [25]. Neural activity was amplified, band-pass filtered, rectified and integrated to create sympathetic neurograms for real-time inspection. Raw unfiltered multifiber nerve activity was recorded at 20 kHz for off-line processing using the algorithm developed in our laboratory to quantify moment-by-moment sympathetic nerve activity [26] (see below). Popliteal artery blood flow velocity was recorded from a 4-MHz Doppler probe (Multidop T2, DWL) at the popliteal fossa of the leg contralateral to the nerve recording. Although changes in vessel diameter over time would confound the estimate of vessel responses, previous work has shown that femoral and popliteal diameters remain unchanged with stimuli that increase flow (calf exercise) [27] and that decrease flow (cold pressor test and hyperemia) [28]. Nonetheless, to ensure that our assumption of constant vessel diameter during handgrip exercise was valid, we compared B-mode ultrasound derived popliteal artery diameters in 7 young subjects during the first and last minutes of exercise (20 measurements for each minute). Handgrip exercise to fatigue did not result in any change in popliteal diameter (1.02±0.08 (SE) vs. 1.02±0.07 cm, *p* = 0.67). Given this data and prior published results, popliteal blood flow velocity was used as a reliable, within-individual surrogate for blood flow to the muscle. However, basal blood flow to the muscle is generally proportional to the tissue volume; as a result, inter-individual differences in body size and composition may confound assessment of sympathetic neurovascular transduction, and so it is necessary to normalize blood flow to account for differences in tissue volume. Therefore, we normalized the blood flow velocity for each individual to the flow velocity during supine rest. This normalization does not impact the goodness-of-fit of neurovascular transduction estimates (described below), but allows us to accurately and reliably capture changes in blood flow while accounting for inter-subject differences in basal flow velocity. All signals were digitized at 20 kHz and stored (PowerLab, ADInstruments) for subsequent off-line analysis.

We used isometric handgrip exercise as a physiologic stimulus to assess sympathetic neurovascular transduction. Isometric handgrip exercise elicits a highly reproducible pressor response mediated by progressive increases in muscle sympathetic nerve activity [29]. Progressive increases in blood pressure drive overall blood flow, whereas those in sympathetic activity cause vasoconstriction to decrease blood flow to non-exercising muscle. As a result of these opposing influences, blood flow to non-exercising muscle remains constant or changes slightly, depending on the artery [30]. Therefore, increasing sympathetic activity during handgrip exercise is causally related to leg blood flow in a feed-forward manner [31]. Moreover, sympathetic activity traverses a physiologic range from resting to very high levels [12], [31]–[33], without any change in cutaneous vascular conductance [34], [35].Thus, the effect of sympathetic activity across a wide range can be explored while avoiding potential confounding factors, such as modulation of sympathetic outflow due to baroreflex feedback and/or alteration of blood flow due to cutaneous vasodilation. Following instrumentation, each subject’s maximal voluntary handgrip force was determined from at least three maximal contractions on a handgrip dynamometer. After a 5-minute resting baseline, subjects performed sustained isometric handgrip exercise to fatigue. During the exercise, target force was displayed on a computer monitor and set at 35% of the maximal voluntary handgrip force. Subjects were provided continuous visual and auditory feedback from the investigators to ensure maintenance of target force until exhaustion, which was defined as a decrease in handgrip force of >10% below the target for >2 sec despite verbal encouragement and the attainment of a maximal perceived exertion.

### Data Analysis

Raw multifiber muscle sympathetic nerve activity was analyzed using the algorithm recently developed and validated in our laboratory [26]. This analysis involves explicit steps to remove noise and movement artifacts and to identify nerve firings based on the statistical properties of background noise. The result is a sympathetic recording with minimal noise that can be quantified as spikes (i.e., nerve firings) per second or per beat. We have previously shown that this algorithm provides an accurate representation of sympathetic activity with greater temporal resolution than sympathetic burst identification [26]. This is important because vasoconstrictor responses may relate not only to the rate of sympathetic firing, but also to the pattern of firing that may not be reflected in the integrated neurogram [36], [37]. Thus, the most accurate representation of sympathetic nerve activity is desirable for reliable characterization of regional sympathetic neurovascular transduction.

After quantification of sympathetic activity, we derived beat-by-beat regional vascular resistance (the ratio of arterial pressure to flow) or conductance (the ratio of flow to arterial pressure). To account for both moment-by-moment and longer term regional sympathetic neurovascular control, we explored the relation of vascular resistance or vascular conductance to sympathetic nerve activity in two ways. First, we averaged resistance or conductance and sympathetic nerve activity over each 30 seconds of handgrip exercise for each individual, and explored each individual’s relationships between averaged values. This is analogous to how this type of data has been explored previously [12], [35], [38]. Second, we used beat-by-beat resistance or conductance and sympathetic activity, and explored each individual’s relationship using the autoregressive model:
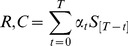
(1)


This examines the beat-by-beat effect of sympathetic activity (*S*) on resistance (*R*) or conductance (*C*) in a time-dependent manner with *T* seconds time lag. We used Akaike’s information criterion [39] to find the best time lag that maximized the goodness-of-fit for each individual.

**Figure 1:**
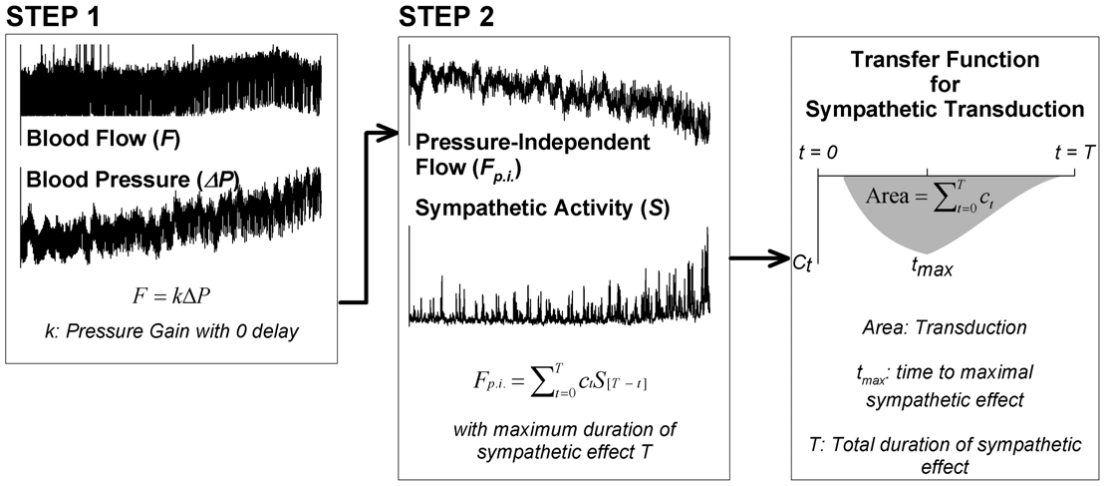
Schematic of the sympathetic neurovascular transduction assessment via an adaptation of Poiseuille’s relation between pressure, sympathetic activity, and flow. We assume that pressure drives flow within the same beat, and fit blood pressure to flow (step 1). The estimated parameter (*k*) provides an estimate for the gain of blood pressure, analogous to admittance (i.e., the inverse of impedance). Subsequently, we fit sympathetic activity to the remaining residuals in flow (pressure-independent flow; step 2). The estimated parameters (*c_t_*) provide values for the time course of regional neurovascular transduction while the total area under the transfer function between sympathetic activity and blood flow provides the regional sympathetic neurovascular transduction estimate (third panel).

We also derived neurovascular transduction from the relationship between arterial pressure, sympathetic nerve activity, and blood flow for each subject. To achieve this, we used Poiseuille’s Law of Flow:
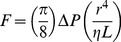
(2)where π/8 is constant, Δ*P* is the change in pressure during the handgrip exercise and the term *r^4^*/(η*L*) provides an estimate of vascular transduction [40]. To derive sympathetic neurovascular transduction explicitly, we assumed that pressure drives flow within the same beat, whereas sympathetic activity affects vessel response over a longer period. Thus, taking the time course of sympathetic effect into account, the adaptation of Poiseuille’s Law of Flow (equation 2) was re-written as



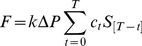
(3)That is, changes in blood pressure (Δ*P*) effect blood flow (*F*) within the same beat, and sympathetic activity (*S*) affects vessel response via a time-dependent transfer function equivalent to an autoregressive model (i.e., the term ∑*c_t_ S_[T – t]_* ) [41]. For ease of implementation and interpretation, we first fit blood pressure to flow using a simple linear equation via ordinary least squares (Step 1 in Figure 1). This accounted for the effect of blood pressure on flow within a beat, and the estimated parameter (*k* in Figure 1) provided an estimate for the gain of blood pressure for each subject (i.e., change in blood flow due to change in blood pressure for each beat). Blood pressure gain is analogous to beat-by-beat ‘admittance’ (i.e., the inverse of impedance), which is a measure of how easily pressure can drive blood flow within a beat. In contrast to arterial pressure, sympathetic activity affects vessel responses over a 10–15 second period [6], [7]. Thus, we subsequently fit sympathetic activity to the remaining residuals in flow (pressure-independent flow; see Step 2 in Figure 1). The estimated parameters (*c_t_*) for each time point *t* provided a transfer function between sympathetic activity and blood flow for each individual while the total area under the transfer function (i.e., the sum of all parameters *c_t_*) provided the sympathetic neurovascular transduction estimate (Figure 1).

All models were estimated using beat-by-beat signals down-sampled to 4 Hz and via custom-written software in Matlab (Mathworks, Natick, MA). We used Akaike’s information criterion [39] to maximize the goodness-of-fit of the models. Note that the time-dependent relationship between resistance (or conductance) and sympathetic activity (equation 1) and Poiseuille’s relation (equation 3) include comparable number of parameters. This ensures that any difference between their ability to explain the data cannot be attributed to over-parametrization. To determine the reproducibility of regional neurovascular transduction estimated via the Poiseuille relation, we used data from five young volunteers who performed two separate handgrip exercises. (Three performed handgrip exercise twice, separated by ∼40 minutes within the same experimental session, and two participated in the study twice in separate sessions approximately two months apart.) Though the changes in arterial pressure, sympathetic nerve activity, and blood flow during handgrip exercise are progressive, the transfer function we used to determine regional neurovascular transduction is not impacted by this form of nonstationarity. The nonstationarity that would confound our estimates is a change in the relationship that occurs over the period of measurement [42]. However, the duration of handgrip exercise was unrelated to sympathetic neurovascular transduction (see below), indicating that the relationship is stationary (i.e., it does not depend on time or overall duration).

### Statistics

The variance explained by different approaches (i.e., *R^2^*) was *r*-to-*z* transformed to ensure normality [43], and compared via one-way repeated measures ANOVA across different approaches (resistance and conductance estimated from 30-sec average values, resistance and conductance estimated from autoregressive model – equation 1, and assessment of regional transduction via the Poiseuille relation – equation 3) followed by Tukey’s HSD post-hoc test. However, *R^2^* values are reported without transformation for ease of interpretation. To assess reproducibility of the Poiseuille relation, we used Lin’s concordance coefficient [44] to test the null hypothesis (H_0_) that the blood pressure gain and neurovascular transduction estimated across two separate handgrip trials are not in agreement. Lin’s concordance coefficient is based on Pearson’s correlation coefficient (a measure of variation), but includes a bias correction term that takes systematic deviations into account. Furthermore, it is a stronger statistical test compared with least-squares linear regression, since it takes both the intercept and the slope into account simultaneously. All hemodynamic variables were normally distributed (Shaprio-Wilk test). Therefore, resting differences in age groups were compared via one-way ANOVA, and differences in hemodynamic responses to handgrip exercise across age groups were compared via two-way repeated measures ANOVA, using age group and exercise as independent factors (younger, <30 years vs. older, >40 years and rest vs. last 30 seconds of handgrip exercise). Blood pressure gain, sympathetic neurovascular transduction, and its time course (time to maximum effect – t_max_ and total duration of effect – *T*) obtained from the Poiseuille relation (equation 3) were compared between age groups using one-way ANOVA. The level of significance was set at *p*<0.05. All data are presented as mean ± standard error.

## Results

Volunteers sustained isometric handgrip exercise for an average of 291±26 seconds. As expected, handgrip exercise elicited significant increases in heart rate (ΔHR: 20±3 bpm), systolic and diastolic pressures (ΔsBP: 43±5 mmHg, ΔdBP: 32±4 mmHg), and sympathetic activity (ΔSNA: 715±88 spikes/min) (*p*<0.05 for all variables). Vascular resistance increased by approximately 50% (from 12.1±1.4 to 18.4±1.7; *p*<0.05), and vascular conductance decreased by approximately 30% (from 0.12±0.02 to 0.08±0.01; *p* = 0.07) by the end of handgrip exercise. Exercise did not affect blood flow velocity significantly (9.5±1.2 cm/s at rest and 8.8±1.4 cm/s at the end of exercise; *p* = 0.15). Hence, hemodynamic responses to handgrip exercise were prototypical.

**Figure 2:**
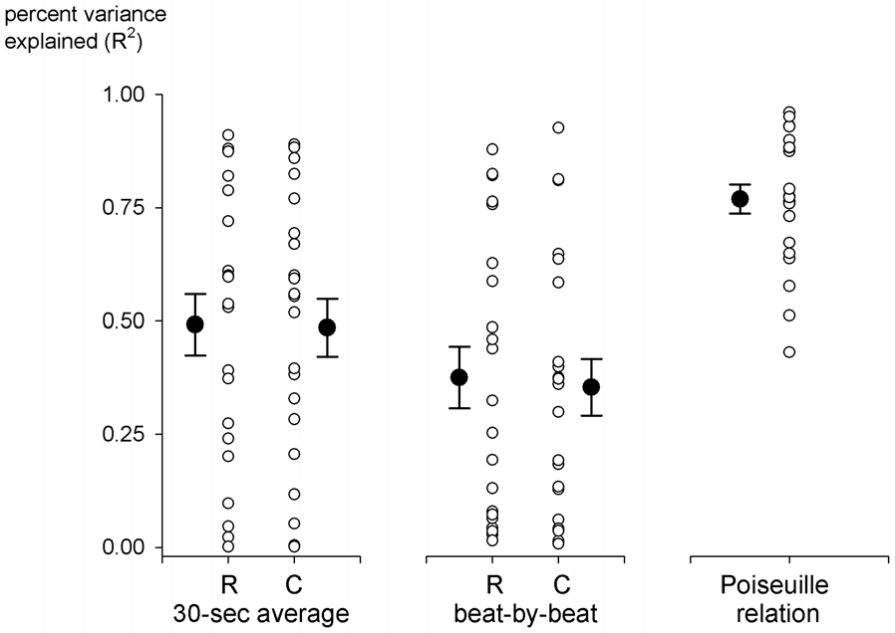
Percent variance explained (*R^2^*) by different approaches to sympathetic neurovascular transduction.

The average relationship between increasing sympathetic activity and vascular responses was good when it was assessed over 30-second averages (resistance, *R^2^* = 0.49±0.07; conductance *R^2^* = 0.49±0.06) but lesser when unaveraged and incorporating time lags (via equation 1; resistance, *R^2^* = 0.38±0.07; conductance, *R^2^* = 0.35±0.06). However, regardless of the approach, the *R^2^* ranged widely across subjects, from 0.01 to 0.91, and was less than 0.50 for half of the individuals (Figure 2). Thus, though these relations were adequate to estimate sympathetic neurovascular transduction in a number of subjects, in at least a third of the subjects, these relations provided relatively weak estimates (*R^2^*<0.33). In contrast, assessment of sympathetic neurovascular transduction from the Poiseuille relation (equation 3 and Figure 1) consistently captured the majority of this relationship. The overall *R^2^* (0.77±0.03) was significantly higher (ANOVA; *p*<0.01) in all but two individuals, and its variability across individuals was markedly lower (Figure 2). In fact, only one relation fell below 50% of explained variance (*R^2^* = 0.43). Across individuals, the *R^2^* was not related to hemodynamic and nervous responses to exercise or to exercise duration (*p*>0.5 for all comparisons).

**Figure 3:**
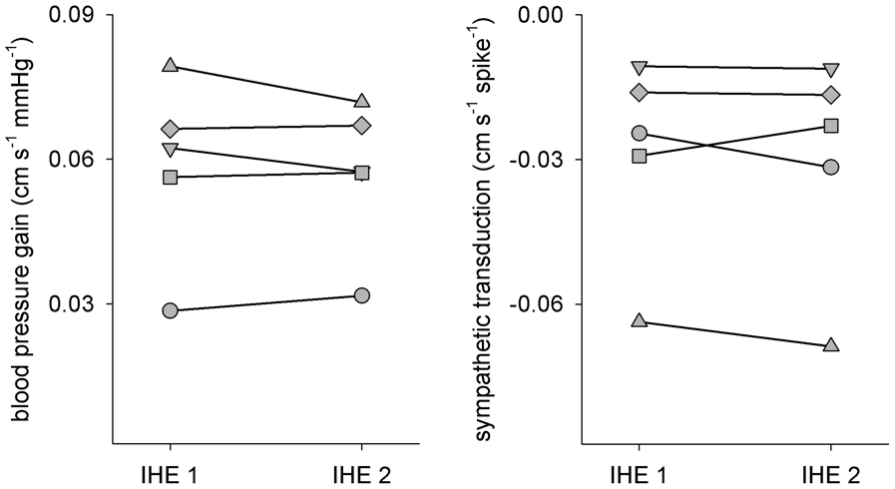
Reproducibility of blood pressure gain and sympathetic neurovascular transduction assessed via the Poiseuille relation. IHE 1 and IHE 2 denote separate isometric handgrip exercises. Each subject is denoted by a different symbol.

**Figure 4:**
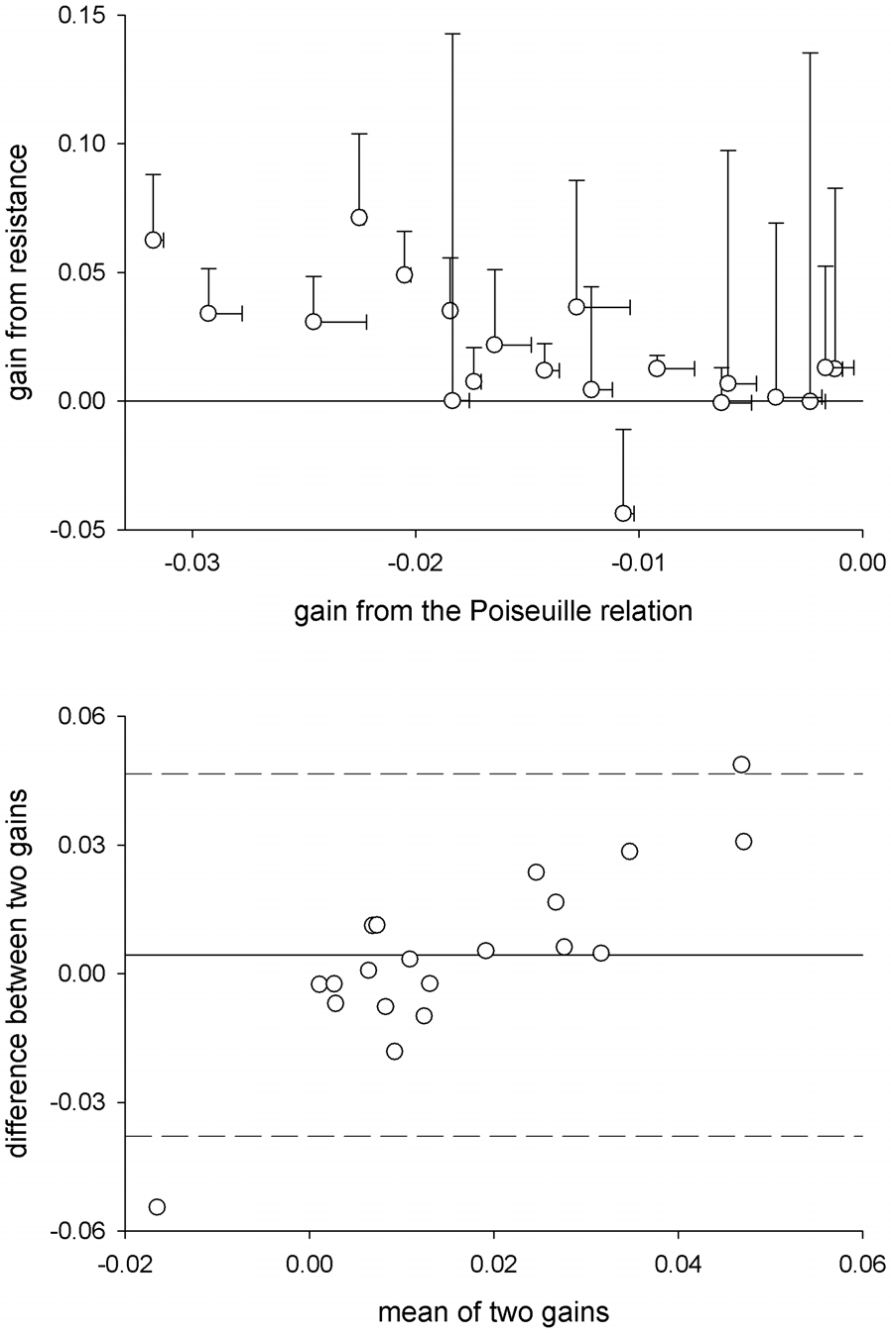
Relation between gain derived from resistance and gain derived from the Poiseuille relation between pressure, sympathetic activity, and flow. Upper panel shows the relation between both gains (linear regression, *R^2^* = 0.37, *p*<0.05; bars show the standard error of the gain relations). Bottom panel shows the Bland-Altman assessment of agreement between the absolute magnitudes of both gains.

The Poiseuille relation also provided estimates of blood pressure gain and sympathetic neurovascular transduction that were reproducible across different study sessions (Lin’s concordance coefficient (ρ_c_) = 0.96, probability of agreement (*p*) = 0.79 for blood pressure gain, and ρ_c_ = 0.97, *p* = 0.87 for sympathetic neurovascular transduction; Figure 3). Thus, the Poiseuille relation offers an accurate and reproducible tool to assess sympathetic control of vascular responses. When we compared the gain of the relation between sympathetic activity and vascular resistance or conductance (over 30-sec averages), we found that the conductance gain was not related to regional sympathetic neurovascular transduction assessed via the Poiseuille relation (linear regression, *R^2^* = 0.02, *p* = 0.59) whereas the resistance gain was inversely related to transduction (linear regression, *R^2^* = 0.37, *p*<0.05). Figure 4 shows the relation between the gains derived from resistance and those assessed from the Poiseuille relation. Bars on the upper panel show the standard error of the estimated gain for each individual. It should be noted that the errors associated with the gains derived from resistance were markedly larger in comparison to those assessed from the Poiseuille relation. Gains with large standard errors correspond to those derived when the strength of the relation between sympathetic activity and vascular resistance was weak (i.e., when the *R^2^* was low; see Figure 2). A Bland-Altman assessment of agreement (Figure 4, lower panel) showed a proportional bias: gain derived from resistance was higher when regional sympathetic neurovascular transduction was high, but was lower when it was low. These results suggest that resistance and conductance are not always reliable surrogates for regional vascular responses, whereas the approach to quantify regional neurovascular transduction based on Poiseuille’s Law of Flow provides an unbiased and reproducible methodology to reliably assess feed-forward control of regional vascular responses from moment-by-moment changes in sympathetic activity.

Next, we used a multiple linear regression to determine if mean resting blood flow was related to the mean resting levels of pressure and sympathetic activity, and to the blood pressure gain and neurovascular transduction derived from handgrip exercise. When a step-wise regression was applied, flow was related to mean pressure and regional neurovascular transduction (*R^2^* = 0.40, *p*<0.05), whereas the regression did not show a significant relation of either resistance or conductance gains to resting flow. Thus, the assessment of regional transduction via the Poiseuille relation, but not via derived indices of resistance and conductance, reflects the physiology of resting blood flow control.

Lastly, we determined whether age-related differences in sympathetic neurovascular transduction reflected the (expected) decline in vascular contractile responses. There was no difference between the two age groups (<30 years or >40 years) in terms of the duration of isometric handgrip exercise to fatigue (291±26 vs. 305±47 seconds; *p* = 0.47). Arterial pressure and sympathetic responses to handgrip exercise were also similar between the two groups (younger vs. older, ΔmBP, 43±6 vs. 29±5 mmHg, *p*>0.1; ΔSNA 802±126 vs. 639±123 spike min^−1^, *p*>0.1). Heart rate responses were blunted in older men (ΔHR 29±4 vs. 11±2 bpm, *p*<0.05). Handgrip exercise increased vascular resistance significantly in both age groups (Δresistance 8.3±2.0 in younger and 5.4±1.4 in older). Vascular conductance was reduced at the end of exercise in both groups (Δconductance 0.06±0.01 in younger and 0.02±0.01), though this reduction was smaller in older individuals (*p*<0.05).

**Figure 5:**
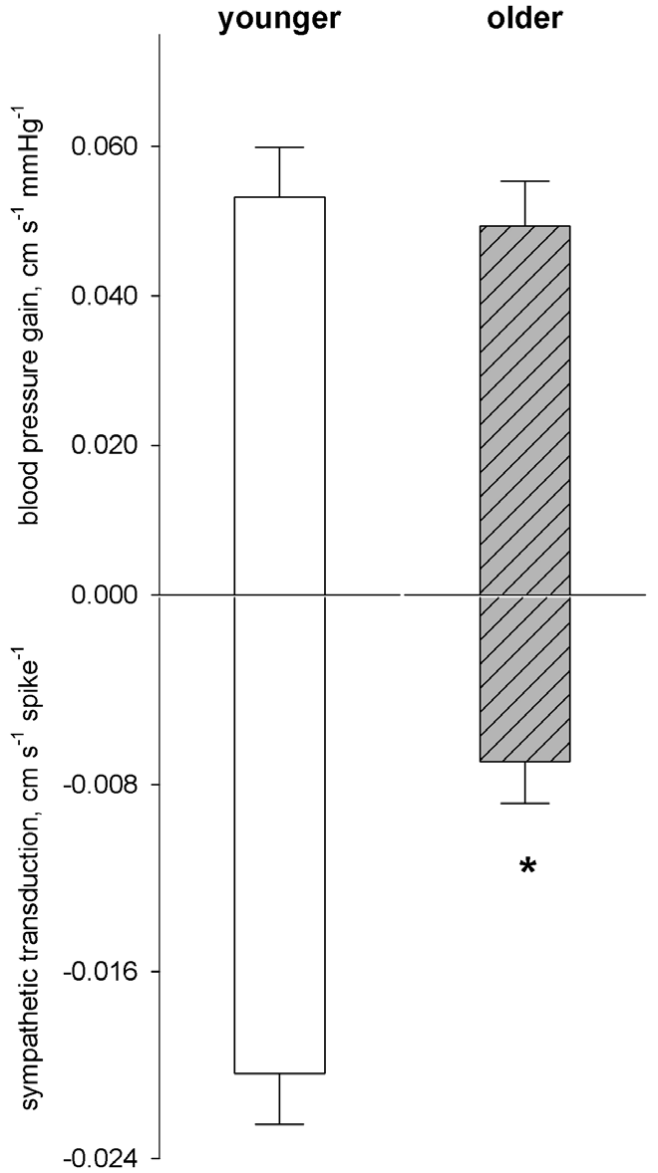
Gain relations between blood pressure and blood flow and between sympathetic activity and blood flow (sympathetic neurovascular transduction) assessed via the Poiseuille relation. *p<0.05.

**Figure 6:**
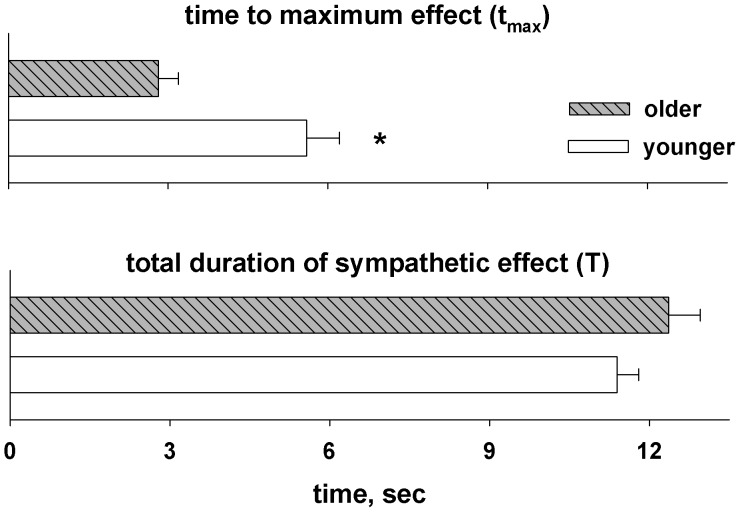
Time course of sympathetic effects. *p<0.05.

When *R^2^* was separated by the two age groups, values were slightly higher in younger than older men (0.84±0.04 vs. 0.71±0.04; *p* = 0.03). The gain relation between arterial pressure and blood flow was not different between age groups (0.053±0.007 cm s^−1^ mmHg^−1^ vs. 0.049±0.006; *p* = 0.66; Figure 5). However, regional sympathetic neurovascular transduction was almost three-fold lower in older men (−0.020±0.002 vs. −0.007±0.002 cm s^−1^ spike^−1^; *p*<0.01; Figure 5). This suggests a marked reduction in the ability of sympathetic outflow to effect vascular responses with age. The time course of sympathetic effect was similar in terms of its total duration (*T*: 11.4±0.4 vs. 12.4±0.6 seconds; *p*>0.1) but the time to its maximal effect was shorter in older men (t_max_: 5.6±0.6 vs. 2.8±0.4 seconds; *p*<0.01; Figure 6). Thus, while the total duration of sympathetic vasoconstrictor effect (that is, the time from sympathetic firing to the end of vascular response) was similar, regional vascular responses to sympathetic outflow were attenuated (i.e., diminished regional transduction), and were more immediate (i.e., shorter time to maximal effect).

**Figure 7:**
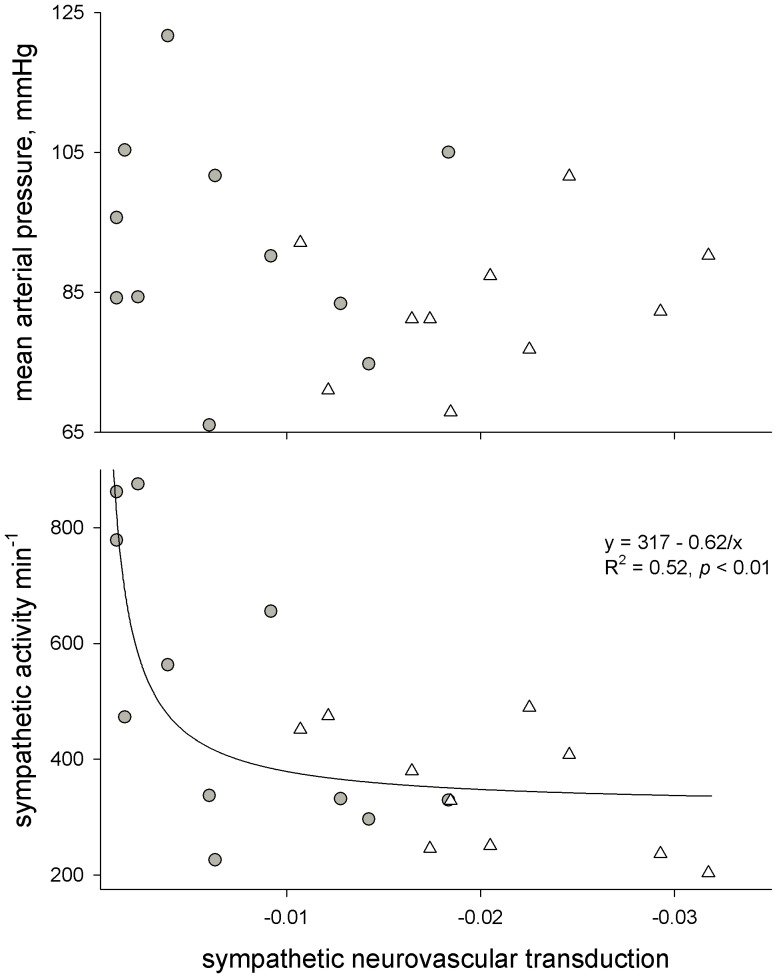
Relation of sympathetic transduction to resting mean blood pressure and resting sympathetic activity. Gray circles denote individuals >40 years of age, and white triangles denote individuals <30 years of age.

There was no relation between regional sympathetic neurovascular transduction and resting heart rate (*R^2^* = 0.01, *p* = 0.75) or resting arterial pressure (*R^2^* = 0.12, *p* = 0.72; Figure 7). To explore the relation between regional sympathetic neurovascular transduction and resting sympathetic activity, we first applied a Granger causality test [45] across all individuals to determine the most likely independent variable. The Granger test indicated that the probable independent variable was regional sympathetic neurovascular transduction (*p*<0.05). There was a modest linear relation between transduction and resting sympathetic activity in both age groups (*R^2^* = 0.26, *p* = 0.07 in younger vs. *R^2^* = 0.36 in older; *p* = 0.03). However, across all individuals, the relation of resting sympathetic nerve activity to regional sympathetic neurovascular transduction was better than in either group alone (*R^2^* = 0.38, *p*<0.01). The relation did not appear to be simply linear (Figure 7), therefore, we applied a basic curvilinear function with the same number of parameters as a linear regression (*y = a+b/x*). This showed a closer relationship between greater resting sympathetic activity and lower sympathetic transduction (*R^2^* = 0.52, *p*<0.01). These results suggest that lesser sympathetic transduction (with increasing age) may lead to greater resting sympathetic activity.

## Discussion

Though our results show that vascular resistance and conductance reflect ∼40–50% of the relation between increasing sympathetic nerve activity and regional vascular responses during isometric handgrip exercise, the strength of the relation can be highly variable across individuals. Thus, these indices are not always reliable surrogates for regional vascular responses. This may not be surprising given the high sensitivity of a given ratio to the variability in its denominator. Small changes in the denominator (i.e., flow or pressure) result in a marked changes in the ratio (i.e., resistance or conductance). This may also explain why assessment of regional sympathetic neurovascular transduction from a Poiseuille relation between arterial pressure, sympathetic nerve activity, and blood flow consistently captured more than 50% of this relationship in all but one individual. We also found that regional sympathetic neurovascular transduction was lesser in older individuals. Though not a universal finding, this agrees with the preponderance of existing data. Thus, assessment of regional sympathetic neurovascular transduction from a Poiseuille relation between pressure, sympathetic activity, and flow may provide a foundation to further explore physiological and pathological differences in sympathetic neurovascular transduction in humans.

The relatively high variability in the relation between sympathetic nerve activity and regional vascular resistance and conductance may reflect the inherent susceptibility of ratios to changes in the numerator and the denominator. Even a small change in either can result in a substantial change in an observed ratio. As a result, small, random beat-by-beat fluctuations in arterial pressure and blood flow may lead to a large variability in resistance or conductance. This can be avoided by using mean values to average out beat-by-beat fluctuations. However, averaging misses the time course at which sympathetic activity effects vascular responses. Thus, we surmised that an examination of the sympathetic activity effect on resistance or conductance in a time-dependent manner would account for the time course and be a more robust analysis. In contrast to our expectation, this second approach resulted in a weaker relationship with equally high variability. This suggests that the advantage of accounting for the time course of vascular responses may be impeded by the large beat-by-beat variability in resistance and conductance (due to small fluctuations in pressure or flow). Moreover, creating a single variable from arterial pressure and blood flow implicitly assumes that sympathetic activity has its effects with the same time course as blood pressure. However, arterial pressure drives flow primarily within the same beat whereas animal studies suggest that sympathetic activity affects vessel responses over a 10–15 second period [6], [7].

Another approach to the relation between sympathetic nerve activity and regional vascular responses would be to account for the different time courses of the two effectors (arterial pressure and sympathetic nerve activity) on the outcome (changes in blood flow). In addition, concurrent beat-by-beat measures of arterial pressure, sympathetic activity, and regional flow obviate the need to rely on a ratio. Therefore, it may not be surprising that we found that adapting Poiseuille’s relation to assess regional sympathetic neurovascular transduction provided the best fit for the linked changes in pressure, sympathetic activity, and regional flow. This improvement cannot be attributed merely to more parameters used since we utilized a similar model with a comparable number of parameters when allowing for time course effects in resistance and conductance. In fact, allowing for time course effects resulted in a *weaker* relationship of sympathetic nerve activity to resistance and conductance compared to 30 second averages. Thus, the approach to quantify regional neurovascular transduction based on a well-established physiological principle (Poiseuille’s Law of Flow) may provide a methodology to reliably assess feed-forward control of regional vascular responses from moment-by-moment changes in sympathetic activity. Moreover, we found that this approach provided reproducible measures that allow assessment of both the time course and the strength of regional sympathetic neurovascular transduction.

Our results show that peak vascular responses to sympathetic outflow occur more quickly and that sympathetic neurovascular transduction is attenuated in older (>40 yrs) compared to younger men. Lesser time to peak vascular responses may simply result from elevated basal sympathetic activity with age. Greater tonic sympathetic activity may relate to greater tonic constriction, resulting in more rapid attainment of peak vascular response. This appears to be in contrast to an earlier study [46] that explored beat-by-beat modulation of arterial pressure by single sympathetic bursts at rest and observed that the peak effect of a single sympathetic burst on arterial pressure occurs later in older individuals compared to younger. However, it is important to note that observations at rest can be misleading due to potential age-related differences in other factors that affect beat-by-beat blood pressure, such as feedback cardiac baroreflex control. (In fact, we used a sympathoexcitatory physiologic stimulus to avoid this confound.).

The decline in transduction is consistent with prior *in vivo* [19], [20] and *in vitro* [21], [22] studies that showed a decline in regional vascular contractile responses to endogenous norepinephrine release with age. We further observed that transduction across all our healthy men was related to resting sympathetic activity. This relationship was not simply linear, but showed a steeper relation as transduction became lower. Within the range of transduction observed in the younger individuals, sympathetic activity remained within a relatively narrow range. But when transduction was below this range, resting sympathetic activity increased as a function of lower transduction. This reciprocal inter-relationship may reflect a compensatory response; as the ability of sympathetic activity to effect regional vasoconstriction declines, sympathetic activity may increase to maintain support of arterial pressure. This would explain the lack of relationship of arterial pressure to transduction. This may seem to stand at odds with data suggesting that resting sympathetic outflow does not relate to systemic vascular resistance in older men [18]. However, three points should be noted. First, the linear relation between regional sympathetic neurovascular transduction and resting sympathetic activity was modest when examined separately for younger and older men, and stronger when examined across all individuals. Second, the relation was not simply linear; a curvilinear function provided the most robust reciprocal inter-relationship between the decline in regional sympathetic neurovascular transduction and the increase in resting sympathetic activity. Third, relations of resting sympathetic activity to whole body flow (i.e., cardiac output) and systemic resistance may not be strictly comparable to regional vascular responses; that is, regional (leg) blood flow control makes up only (some) part of systemic hemodynamic control.

Presumably, the chronic elevation in sympathetic nervous outflow with advancing age is to preserve homeostatic control of global cardiovascular function. For example, both stroke volume [47] and cardiac output [48] are reduced with age, and the elevation in resting sympathetic outflow may result from a centrally mediated sympathoexcitation to maintain appropriate blood pressure. This would suggest that resting sympathetic activity and cardiac output should be inversely related. However, though this relation may hold for young men [49], it is fully absent in young women [50] and older men [18]. In fact, one study found that in older men without any evidence of lower cardiac output or increased arterial pressure, tonic sympathetic activity was fifty percent higher than in young men [18]. Furthermore, we have previously shown that baroreflex control of vascular sympathetic activity is not impacted by age, and does not relate to resting sympathetic outflow [51]. Therefore, the age-related chronic elevation of sympathetic nervous outflow does not appear to relate to altered hemodynamic control.

Alternatively, energy intake and total body fat increases with advancing age in the general population [52], and a centrally mediated sympathoexcitation may serve to stimulate energy expenditure in response to increased adiposity with age. For example, the sympathetic nervous system is activated with excessive energy intake [53], [54], and lipid infusion increases sympathetic outflow as much as 40% [55], whereas weight loss reduces sympathetic outflow [56]. Thus, it would seem that increased body adiposity and subsequent increase in circulating leptin and insulin with advancing age leads to central sympathoexcitation [57], [58]. However, aging has no effect on circulating leptin level [59] or insulin resistance [60] independent of body habitus, and we have previously found that resting sympathetic outflow in older master’s athletes is comparable to that in age-matched sedentary individuals despite a substantially lower body adiposity [51]. Therefore, centrally driven sympathoexcitation due to an alteration in metabolic demand cannot fully account for the elevated sympathetic outflow with advancing age.

Thus, the age-related elevation in sympathetic nervous outflow cannot be simply explained by a centrally mediated sympathoexcitation in response to a reduction in cardiac output, a shift in baroreflex control of vascular sympathetic outflow, or a change in energy expenditure. Its origin may reside in the interrelationship between centrally driven sympathoexcitation and a decline in the ability of sympathetic outflow to effect peripheral vascular responses, i.e., a decline in peripheral sympathetic neurovascular transduction. It can be argued that this decline in neurovascular transduction is merely a consequence of adrenergic receptor desensitization in the face of centrally mediated sympathoexcitation with advancing age. Though this is a reasonable postulate, if true, the extent of decline in neurovascular transduction should be comparable to the elevation in sympathetic outflow. Yet, the age-related decline in transduction (as well as the increase in vascular resistance) appears to be in excess of the increase in sympathetic nervous outflow. For example, pervious work shows that despite an almost two-fold increase in sympathetic nerve activity [17], [51] and arterial norepinephrine concentration [61], systemic and regional resistance is elevated by only ∼45% or less in older individuals [17], [20], [62]. In addition, our *in vivo* measures also suggest that functional declines in sympathetic neurovascular transduction are markedly greater; neurovascular transduction was ∼70% lower whereas sympathetic nerve activity was only ∼50% higher in our middle-aged compared to young volunteers. These striking differences suggest that though some of the decline in vascular responsiveness may be due to adrenergic receptor desensitization, there may be a decline in peripheral sympathetic neurovascular transduction with advancing age independent of elevated sympathetic outflow.

Declines along the cascade of events that link nervous firing to vascular response other than alpha adrenergic responsiveness may account for these differences. For example, NO increases arterial elasticity [63], [64], and vessel elasticity is directly related to the ability of electrical, pharmacological, and reflex activation of the sympathetic system to effect vascular responses [65], [66]. Therefore, NO availability has a prominent effect on adrenergically mediated vasoconstriction, and thus, on neurovascular transduction. Disruption of post-translational intracellular pathways [67] and increased oxidative stress due to greater production of reactive oxygen species with advancing age [68], [69] have adverse effects on endothelial nitric oxide synthase activity and NO production. As a result, global [70] as well as vascular [68], [69] NO release is attenuated with advancing age independent of other factors. Therefore, it is reasonable to suggest that the attenuation in NO bioavailability with advancing age is a precipitating factor in the decline in neurovascular transduction, independent of changes in sympathetic outflow. Nonetheless, our aim was not to tease apart these various contributors, but rather our work lays a foundation for future work to consider the role of various contributors to the age-related differences in sympathetic neurovascular transduction.

In summary, our results show that though both resistance and conductance capture ∼50% of the relationship between sympathetic activity and regional vascular responses, their utility may be limited by the high variability in the strength of this relationship across individuals. In contrast, assessment via a Poiseuille relation captured ∼75% of the relationship between arterial pressure, sympathetic nerve activity, and blood flow consistently across all individuals. In addition, this approach provided a measure of regional sympathetic neurovascular transduction that was reproducible within individuals and consistent with earlier data. Thus, this approach may provide a tool to further explore the physiology of regional vascular control, and may be particularly useful in exploring pathophysiological alterations in sympathetic vascular control.
